# Recombinant *Mycobacterium paragordonae* Expressing SARS-CoV-2 Receptor-Binding Domain as a Vaccine Candidate Against SARS-CoV-2 Infections

**DOI:** 10.3389/fimmu.2021.712274

**Published:** 2021-08-27

**Authors:** Byoung-Jun Kim, Hyein Jeong, Hyejun Seo, Mi-Hyun Lee, Hyun Mu Shin, Bum-Joon Kim

**Affiliations:** ^1^Department of Microbiology and Immunology, College of Medicine, Seoul National University, Seoul, South Korea; ^2^Department of Biomedical Sciences, College of Medicine, Seoul National University, Seoul, South Korea; ^3^Liver Research Institute, College of Medicine, Seoul National University, Seoul, South Korea; ^4^Cancer Research Institute, College of Medicine, Seoul National University, Seoul, South Korea; ^5^Seoul National University Medical Research Center (SNUMRC), Seoul, South Korea; ^6^BK21 FOUR Biomedical Science Project, Seoul National University College of Medicine, Seoul, South Korea; ^7^Interdisciplinary Program in Cancer Biology, College of Medicine, Seoul National University, Seoul, South Korea; ^8^Wide River Institute of Immunology, Seoul National University, Hongcheon, South Korea

**Keywords:** SARS-CoV-2, COVID-19, vaccine, *Mycobacterium paragordonae*, receptor-binding domain (RBD)

## Abstract

At present, concerns that the recent global emergence of SARS-CoV-2 variants could compromise the current vaccines have been raised, highlighting the urgent demand for new vaccines capable of eliciting T cell-mediated immune responses, as well as B cell-mediated neutralizing antibody production. In this study, we developed a novel recombinant *Mycobacterium paragordonae* expressing the SARS-CoV-2 receptor-binding domain (RBD) (rMpg-RBD-7) that is capable of eliciting RBD-specific immune responses in vaccinated mice. The potential use of rMpg-RBD-7 as a vaccine for SARS-CoV-2 infections was evaluated in *in vivo* using mouse models of two different modules, one for single-dose vaccination and the other for two-dose vaccination. In a single-dose vaccination model, we found that rMpg-RBD-7 *versus* a heat-killed strain could exert an enhanced cell-mediated immune (CMI) response, as well as a humoral immune response capable of neutralizing the RBD and ACE2 interaction. In a two-dose vaccination model, rMpg-RBD-7 in a two-dose vaccination could also exert a stronger CMI and humoral immune response to neutralize SARS-CoV-2 infections in pseudoviral or live virus infection systems, compared to single dose vaccinations of rMpg-RBD or two-dose RBD protein immunization. In conclusion, our data showed that rMpg-RBD-7 can lead to an enhanced CMI response and humoral immune responses in mice vaccinated with both single- or two-dose vaccination, highlighting its feasibility as a novel vaccine candidate for SARS-CoV-2. To the best of our knowledge, this study is the first in which mycobacteria is used as a delivery system for a SARS-CoV-2 vaccine.

## Introduction

To stop the current COVID-19 pandemic caused by severe acute respiratory syndrome coronavirus 2 (SARS-CoV-2) (1–4), there is a need to develop an effective vaccine to elicit a strong protective immune response against SARS-CoV-2 and its variants that are compromising vaccine efficacy. Most vaccines in current use or those being developed have been focused on enhancing the production of neutralizing antibodies (5–7). However, several lines of evidence indicate that to protect the mutated variants being generated worldwide, a SARS-CoV-2 vaccine for eliciting CD4 and CD8 T cell-mediated immune responses as well as neutralizing antibodies should be developed (8–12).

Due to its excellent vaccine adjuvant properties, which are particularly capable of enhancing T cell-mediated immune responses, *Mycobacterium* spp. including *Mycobacterium bovis* bacille Calmette-Guérin (BCG) or *Mycobacterium smegmatis* is being studied as a live vaccine delivery vector to confer protection against various infectious pathogens, including human immunodeficiency virus type 1 (HIV-1) (13–16), measles virus (17), *Leishmania* (18), rodent malaria (19) and even cancer (20–22). Notably, *Mycobacterium paragordonae* (Mpg), a putative tuberculosis vaccine candidate (23), has been shown to elicit more robust immune responses in vaccinated mice studied as tuberculosis (24) or cancer vaccine models (25) than BCG. Moreover, this trend is also true in recombinant Mpg-expressing HIV-1 p24 (26), suggesting the merit of using rMpg as a vaccine delivery system.

Therefore, to develop a SARS-CoV-2 vaccine capable of eliciting both humoral and cell-mediated immune responses, in this study, we sought to construct a novel recombinant *Mycobacterium paragordonae* expressing the SARS-CoV-2 receptor-binding domain (RBD) (rMpg-RBD). Its potential use as a vaccine against SARS-CoV-2 was evaluated through two *in vivo* experiments, one for a single-dose vaccination model and the other for a two-dose vaccination model.

## Materials and Methods

### Construction of pMV306 Vector for Expression of the SARS-CoV-2 RBD Antigen

To generate a *Mycobacterium*-*E. coli* shuttle vector expressing the SARS-CoV-2 RBD antigen, the heat shock protein 65 gene (*hsp65*) promoter region and DNA sequences of the RBD gene were amplified by overlapping PCR. The *hsp65* promoter region (P_hsp_) was obtained from the genomic DNA of *M. bovis* BCG. The RBD gene was amplified from the synthesized RBD gene (corresponding to amino acids 319-541 of the SARS-CoV-2 spike S1 protein, GenBank no. MN996527) optimized for the codons of *Mycobacterium tuberculosis* (TB) (Bionics, Seoul, Korea). RBD gene codon optimization was performed using the JCat web-based tool (27). The forward primer sequence *hsp65* promoter was 5’ – AAAGCGGCCGCGGTGACCACAACGACGC - 3’ (*Not*I), and the reverse primer sequence was 5’ - CTGCACGCGCATTGCGAAGTGATTCCTCC - 3’. The forward primer sequence for amplifying the RBD gene was 5’ - GGAGGAATCACTTCGCAATGCGCGTGCAG - 3’, and the reverse primer sequence was 5’ – AAATCTAGATTAGAAGTTCACGCACTTGTTCTTC - 3’ (*Xba*I). The amplified *hsp65* promoter region and RBD gene were overlapped by PCR using the *hsp65* promoter forward primer and RBD gene reverse primer. The overlapping P_hsp_ -RBD sequence was digested with *Not*I and *Xba*I (NEB, Massachusetts, USA) and cloned into the *Mycobacterium*-*E. coli* shuttle vector pMV306 with T4 ligase mix (TaKaRa, Kyoto, Japan).

### Generation of a Recombinant Mpg Strain Expressing the SARS-CoV-2 RBD Protein

To generate the recombinant Mpg (rMpg) expressing SARS-CoV-2 RBD, the pMV306-RBD plasmid was electroporated into a competent Mpg using the GenePulser Xcell™ electroporation machine (Bio-Rad, California, USA) under 2.5 kV, 25 μF, and 1,000 Ω conditions. The transformants were plated and selected on Middlebrook 7H10 agar medium (Becton, Dickinson, and Company (BD, New Jersey, USA) supplemented with OADC (BD, New Jersey, USA) containing 100 μg/ml kanamycin for 2 or 3 weeks at 30 °C. The colonies were confirmed by PCR with the *hsp65* promoter forward primer and RBD gene reverse primer to amplify the RBD gene.

### Bacterial Culture and Preparation

Mpg or rMpg-RBD strains were cultured at 30 °C in Middlebrook 7H9 broth (BD) supplemented with 2.5% glycerol, 0.2% Tween-80, and 10% of albumin-dextrose-catalase (ADC; BD, New Jersey, USA) or on Middlebrook 7H10 agar media supplemented with 0.5% glycerol and 10% oleic acid-albumin-dextrose-catalase (OADC; BD, New Jersey, USA) for 2 weeks. In the case of rMpg-RBD cultivation, 100 μg/mL of kanamycin was added in all the media. To obtain single cell suspensions for immunization, cultures in exponential phase were suspended in PBS with 1% Tween 80 (PBS-T) and passed through a 27-gauge needle three to five times. Single cell suspensions were adjusted to 0.2 optical density at 600 nm wavelength (OD_600nm_), serially diluted and colony forming units (CFUs) for immunization or cell infection were enumerated on the Middlebrook 7H10 agar plates (0.2 OD value of bacterial suspension was approximately 4~5 x 10^7^ CFU/ml of Mpg or rMpg-RBD strains).

Also, 1, 5, and 10 passaged rMpg-RBD were used to check the expression of RBD in rMpg-RBD strains. Each passage process was conducted from 7H9 broth (with 100 μg/mL of kanamycin) to 7H9 broth for 2 weeks.

### Mice and Immunization Procedures

Female BALB/c mice (~25 g, 7 weeks old) were purchased (Orient Bio, Seoul, Korea) and used for the experiments. The mice were randomly divided into five or six groups of two or five mice per group. Three types of animal experiments were conducted in this study. To select an rMpg-RBD strain with an enhanced RBD or S1-specific immune response, the mice (*n* = 2~3) were subcutaneously (S.C.) injected twice (two-week interval) with wild-type Mpg or rMpg-RBD strains (-5, -6, and -7) (1 × 10^6^ CFU in 100 μl of PBS/mouse) (**Figure 2A**).

For the two-dose vaccination test, the mice were S.C. immunized with i) wild-type Mpg (two times with two-week intervals), ii) rMpg-RBD-7, iii) rMpg-RBD-7 (two times with two-week intervals), iv) rMpg-RBD-7 prime-RBD protein boosting, and v) RBD protein (two times with two-week intervals). The PBS injection group was used as a negative control group (**Figure 4A**). 1 x 10^6^ CFU of Mpg or rMpg-RBD was vaccinated. RBD protein (V40592-V08B; Sino Biological, Beijing, China; 10 μg/mouse) was injected with alum adjuvant (100 μg/mouse).

For the single-dose vaccination test, the mice were S.C. injected with i) wild-type Mpg, ii) live rMpg-RBD (1 × 10^6^ CFU in 100 μl of PBS/mouse), iii) live rMpg-RBD-7 (1 × 10^7^ CFU in 100 μl of PBS/mouse), and iv) heat-killed rMpg-RBD (1 × 10^6^ CFU in 100 μl of PBS/mouse). For the negative group, PBS was S.C. injected into the mice (**Figure 7A**). Heat-killed rMpg-RBD was prepared by heating the rMpg-RBD strain of 1 × 10^7^ CFU (at 80 °C for 20 minutes), pelleted by centrifugation (13,000 rpm for 2 minutes) and suspended in 1 ml of PBS.

Two or four weeks after the final immunization, the immunized mice were euthanized by CO_2_ inhalation, and their blood, spleen and bronchoalveolar lavage fluid (BALF) samples were obtained and used in immunological assays such as RBD- or S1-specific IFN-γ ELISPOT, cytokine determination, antibody detection in serum or BALF, T cell analyses and neutralizing antibody detection.

### IFN-γ Enzyme-Linked Immunospot (ELISPOT)

Splenocytes from immunized mice (BALB/c) with the Mpg wild type and rMpg-RBD were used to conduct the ELISPOT assay. An ELISPOT plate (PVDF membrane; Millipore, Massachusetts, USA), which was activated using 30% ethanol, was coated with mouse IFN-γ capture antibody (3 µg/mL, Clone: AN-18; Thermo Fisher, Massachusetts, USA) at 4°C overnight.

The plate was washed three times with PBS-T, which was 0.05% TWEEN-20 (Sigma-Aldrich, Missouri, USA) in PBS, and PBS. The cells were blocked with 200 µL of complete RPMI 1640 medium supplemented with 10% fetal bovine serum (Thermo Fisher, Massachusetts, USA) and penicillin-streptomycin (Welgene, Gyeongsan, Korea) at 37 °C for 2 h. After the complete RPMI was discarded, 1 x 10^6^ splenocytes were loaded into each well. In the antigen treatment group, each well was added in duplicate with 5 µg/mL SARS-CoV-2 spike (S1) protein (40591-V08B1; Sino Biological, Beijing, China) or SARS-CoV-2 spike (RBD) protein (40592-V08B; Sino Biological, Beijing, China) in a total volume of 200 µL for stimulation, and in the negative control group, complete RPMI was added to the wells to fill up to 200 µL. In the positive control group, 50 ng/mL phorbol 12-myristate 13-acetate (PMA) (Sigma-Aldrich, Missouri, USA) and 1 µg/mL ionomycin (Sigma-Aldrich, Missouri, USA) were added to the wells to reach a total volume of 200 µL.

After the plate was incubated at 37 °C overnight without rocking, the cells on the plate were discarded and washed with distilled water (one time, for 10 min), PBS-T, and PBS (three times). The wells were incubated with anti-mouse IFN-γ-biotin (3 µg/mL, Clone: XMG 1.2; Invitrogen, Massachusetts, USA) at 4 °C overnight and washed with PBS-T and PBS three times. The wells were incubated with streptavidin-HRP (BD, New Jersey, USA) at RT for 2 h and washed. Spot development was performed using an AEC substrate set (BD, New Jersey, USA), stopped within 10 min, and washed with distilled water. The completely dried plate was read using an ELISPOT reader (AID ELISPOT reader; AID, Baden-Württemberg, Germany) to count the spot forming units (SFUs).

### Determination of RBD Expression Levels in the rMpg-RBD Strain

To determine the RBD expression levels in the rMpg-RBD strain, we conducted Western blot, enzyme-linked immunosorbent assay (ELISA), and real-time quantitative PCR. First, to extract protein from cultured rMpg-RBD, pellet was suspended in B-PER buffer (Thermo Fisher, Massachusetts, USA) supplemented with lysozyme (100 μg/ml), DNase (5 U/ml), and proteinase inhibitor. The suspension was sonicated for 5 min (pulse: 0.3 sec, stop: 0.7 sec) on ice and centrifuged at 13,000 rpm, 4 °C for 15 min. Protein concentration of the supernatant sample was quantified by Quick Start™ Bradford Protein Assay kit (Bio-Rad, California, USA). The expression of RBD in the rMpg-RBD strain was determined using an RBD ELISA kit (ELV-COVID19S1-1; Ray Biotech, Georgia, USA) according to the manufacturer’s instruction. 1, 5 and 10 passaged rMpg-RBD strains were used to confirm the stable expression of RBD.

Total RNA was purified from rMpg-RBD strains or DC2.4 cells infected with Mpg or rMpg-RBD using TRIzol reagent (Invitrogen, Massachusetts, USA). Purified RNA samples were used as templates for real-time quantitative PCR (RT-qPCR) with SensiFAST™ SYBR Lo-ROX One-Step kit (Bioline, London, UK). The primer pairs used to amplify RBD and *hsp65* (internal control) are given in **Supplementary Table S1**. The reaction was carried out with CFX96™ Real-Time PCR detection system (Bio-Rad, California, USA) and the data were analyzed using the Ct (cycle threshold) values.

### Cytokine Determination From Infected-Dendritic Cells

DC2.4 cells, the murine dendritic cell line, were maintained in complete RPMI 1640 medium (Welegene, Gyeongsan, Korea) with L-glutamine (300 mg/L) and NaHCO_3_ (2000 mg/L), complemented with 10% fetal bovine serum (FBS) (Thermo Fisher, Massachusetts, USA), penicillin-streptomycin (100 units/mL and 100 µg/mL) (Welgene, Gyeongsan, Korea), at 37 °C in a humidified atmosphere of 5% CO_2_. The 5 × 10^5^ cells (in a 24-well plate and triplicate) were infected with Mpg (10 M.O.I.; multiplicity of infection) and rMpg-RBD (1 or 10 M.O.I.) in RPMI 1640 medium only complemented with 10% FBS for 4 hours at 37 °C. As a negative control, it was treated with culture medium, and as a positive control, it was treated with 1 µg/mL LPS (Sigma-Aldrich, Missouri, USA). After infection, each well was washed with PBS three times and incubated in the complete RPMI 1640 medium before collecting culture supernatant. The supernatant was used for measuring IL-10 (Invitrogen, Massachusetts, USA) and IL-12p40 (Invitrogen, Massachusetts, USA) using ELISA kits according to the manufacture’s manual.

### Flow Cytometry Assay for DC Maturation

Mpg- or rMpg-RBD-infected DC2.4 cells were detached from each well and blocked with CD16/32 antibody (Bio Legend, California, USA) for 30 min. As a positive control, LPS-treated (1 μg/ml) DC2.4 cells were also used. After being washed with PBS-T, the cells were stained with BV605-conjugated anti-CD86, PE-conjugated anti-CD40, FITC-conjugated anti-MHCII, and APC-conjugated anti-CD80 antibodies (BD, New Jersey, USA) for 30 min on ice and washed three times with PBS. The stained DC2.4 cells were resuspended in FACS buffer (PBS with 1% bovine serum albumin and 1 mM EDTA). The stained DC2.4 cells were analyzed by flow cytometry (BD LSRFortessa). The data were analyzed with FlowJo software (FlowJo, Oregon, USA).

### Serum or BALF Antibody Detection

To detect the serum or BALF antibody levels, serum or BALF samples were collected from the immunized mice following euthanasia *via* CO_2_ inhalation. The 96-well plate was coated with purified RBD or S1 protein (Sino Biological) in 0.05 M carbonate-bicarbonate buffer (pH 9.6) overnight at 4 °C. Serum or BALF samples were added each well and incubated for 2 hours at RT. After washing each well three times with PBS-T, RBD or S1 protein-specific antibodies in serum or BALF samples were detected with mouse IgG2a, IgG1 (BD, New Jersey, USA; 1:1,000 dilution), total IgG (Invitrogen, Massachusetts, USA; 1:1,000 dilution) or IgA (Invitrogen, Massachusetts, USA; 1:1,000 dilution) antibodies. All the detection antibodies were conjugated with horseradish peroxidase (HRP). After final washing, the plates were developed using 3,3’,5,5’-tetramethylbenzidine (TMB) solution (Invitrogen, Massachusetts, USA). Reactions were stopped using 1N sulfuric acid within 5 minutes. The optical density (OD) was determined using a spectrometer at a wavelength of 450 nm.

### SARS-CoV-2 Pseudovirus Neutralization Assay

Pseudotyped SARS-CoV-2 preparation and neutralization assays were performed to detect wild type Mpg or rMpg-RBD-7-induced neutralizing antibodies against pseudotyped SARS-CoV-2 infection as described previously (28). In brief, the plasmids of pNL4-3.luc.R-E- (3418; NIH-AIDS) and pCAGGS encoding SARS-CoV-2 spike glycoprotein S (NR-52310; BEI Resources) were co-transfected into 293T cells. Seventy-two hours after co-transfection, the harvested supernatant media was mixed with PEG-it virus precipitation solution (LV810A-1-SBI) and centrifuged at 1,500 g for 30 min to obtain a lentivirus pellet. Single-use aliquots were stored at -80 °C. Thereafter, the tissue culture infectious dose (TCID_50_) was determined by infections of Huh7, Calu-3, and Vero E6 cells (29). To evaluate the pseudovirus neutralization activity of the mouse serum, an equal volume of ~120 TCID_50_ of pseudotyped SARS-CoV-2 was incubated with diluted serum for 1 h at 37 °C, added to the Huh7 and Calu-3 cells, and then cultured at 37 °C for two days. The cells were lysed using cell lysis buffer (Promega, Wisconsin, USA) and transferred into luminometer 96-well plates. Luciferase substrate (Promega, Wisconsin, USA) was added to the lysates, and the relative luciferase activity was measured using a luminometer (Tecan).

### Live SARS-CoV-2 Neutralization Assay

Wild type Mpg or rMpg-RBD-7-induced neutralizing antibodies against live SARS-CoV-2 infection were detected using the plaque assay as described previously (28). The experiment was conducted in a BSL-3 laboratory according to the guidelines approved by the Institutional Biosafety Committee of Seoul National University (Approval No. SNUIBC-R200427-2). In brief, serum from each immunized mouse was diluted and mixed with the same volume of SARS-CoV-2 (~120 pfu) and incubated at 37 °C for 1 h. Thereafter, 200 μl of the virus-serum mixtures were transferred to pre-plated Vero E6 cells (cells/well) in 24-well plates. Inoculated cells were incubated at 37 °C for two days. Then, Vero E6 cells were fixed with 4% paraformaldehyde and permeabilized with 100% methanol. The cells were incubated sequentially with primary antibodies against the SARS-CoV-2 nucleocapsid (NP) overnight at 4 °C, alkaline phosphatase (AP)-conjugated secondary antibody, and NBT/BCIP. The plaque reduction neutralizing antibody titer (PRNT_50_) was calculated as the highest dilution of serum capable of preventing SARS-CoV-2-induced plaque formation in 50% of that in the Mpg (control) in this study.

### RNA Extraction and RT-qPCR

Total RNA was extracted from live SARS-CoV-2-infected Vero E6 cells using TRIzol reagent (Invitrogen, Massachusetts, USA). The expression level of the target gene was analyzed by quantitative reverse transcription-PCR using the ABI 7500 system (Applied Biosystems, Thermo Fisher, Massachusetts, USA) and specific primers for RNA-dependent RNA polymerase (RdRp) and GAPDH and the SYBR green PCR master mix (Applied Biosystems, Thermo Fisher, Massachusetts, USA). The forward primer sequence of the RdRp gene was 5’–GTGARATGGTCATGTGTGGCGG-3’, and the reverse primer sequence was 5’- CARATGTTAAASACACTATTAGCATA–3’. The forward primer sequence of the GAPDH gene was 5’- GGATTTGGTCGTATTGGG–3’, and the reverse primer sequence was 5’- GGAAGATGGTGATGGGATT-3’.

### IFN-γ Secreting T Cell Analysis

Splenocytes from each immunized mouse were seeded into 96-well plates (1 × 10^6^ cells/well) in complete RPMI-1640 medium and stimulated with RBD protein (Sino Biological, Beijing, China; 5 μg/ml) in a total volume of 200 μl for 20 h. Stimulation with PMA (50 ng/ml) and ionomycin (1 μg/ml) was used as a positive control. To prevent cytokine release, brefeldin A (10 μg/ml) was added, and the cells were cultured for an additional 4 h. Cell surface markers were stained with FITC-conjugated anti-CD3, V500-conjugated anti-CD4 and PE-conjugated anti-CD8 (BD, New Jersey, USA) antibodies. After being fixed with 1% paraformaldehyde (20 min, RT) and permeabilized with 0.1% Triton X-100 (30 min, RT), the cells were stained intracellularly with APC-conjugated anti-IFN-γ (BD, New Jersey, USA) antibody. The stained cells were analyzed using a FACS LSRFortessa instrument (BD, New Jersey, USA) and the FlowJo program.

### Determination of Cytokine Production in Immunized Mice

Splenocytes from the immunized mice were plated at a concentration of 1 × 10^6^ cells/well (96-well microplate, 200 μL volume) in RPMI 1640 medium supplemented with 10% FBS, and purified RBD or S1 protein (Sino Biological, Beijing, China) was added at a concentration of 5 μg/ml for the *in vitro* stimulation. The cell culture supernatants were harvested at 3 days after the stimulation to determine the expression levels of the cytokines IFN-γ, TNF-α, IL-2, IL-10, and IL-12 (Invitrogen, Massachusetts, USA) using ELISA kits.

### ACE2-RBD Binding Inhibition Assay

Using the COVID-19 Spike-ACE2 Binding Assay kit (CoV-ACE2S2-1; Ray Biotech, Georgia, USA), we evaluated whether the antibodies in the serum of mice immunized with Mpg, rMpg-RBD or heat killed rMpg-RBD strains inhibit the binding of ACE2 and RBD. Serum samples from immunized mice were diluted 1:10 to 1:1,000 in PBS, mixed with RBD protein, and then loaded in a 96-well plate coated with ACE2 protein. Following 3 h of incubation at RT, the cells were washed four times with washing buffer, and HRP-conjugated IgG was added to each well for 1 hour at RT. After 3,3’,5,5’-tetramethylbenzidine (TMB) solution was added, and the absorbance was measured at 450 nm with an ELISA reader device (Tecan Sunrise). Measurements were performed in triplicate, and OD values were converted to percent inhibition (the highest OD values as 100% and the lowest OD values as 0%) and plotted with a nonlinear regression curve fit using the PRISM program.

### Statistical Analysis

All the presented data are expressed as the means ± SEM. The significant differences among each experimental group were evaluated by one-way analysis of variance (ANOVA) with Tukey’s *post-hoc* test using GraphPad Prism software (GraphPad, USA). Differences were considered significant at a probability value of less than 0.05.

## Results

### Construction of Recombinant *Mycobacterium paragordonae* Expressing SARS-CoV-2 Receptor Binding Domain (rMpg-RBD) as a Vaccine Candidate for SARS-CoV-2 and Selection of an rMpg-RBD Strain With Strong Immune Responses in Vaccinated Mice

**Figure 1:**
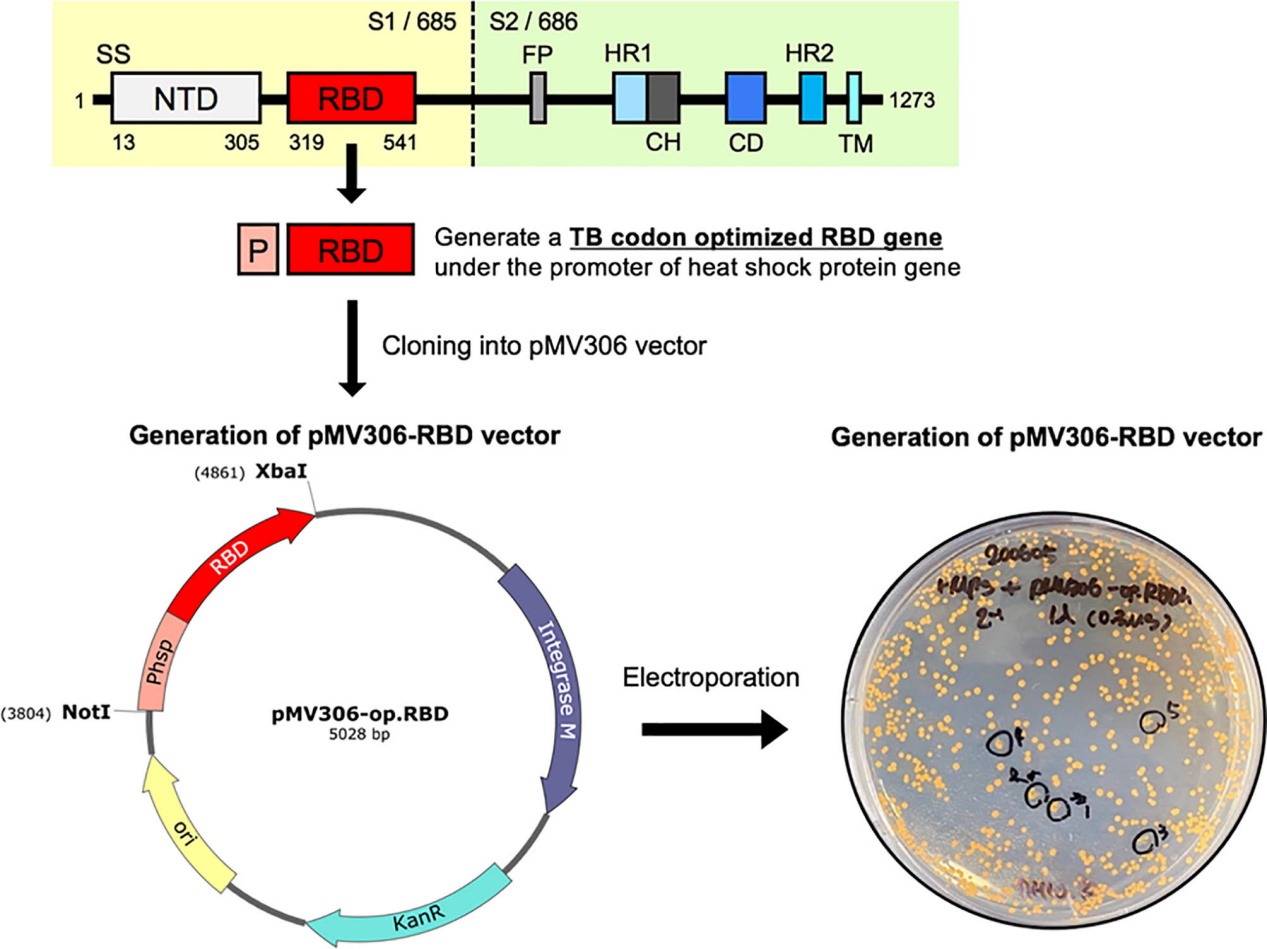
Schematic presentation showing the construction of recombinant *Mycobacterium paragordonae* expressing SARS-CoV-2 receptor binding domain (rMpg-RBD). A TB-optimized SARS-CoV-2 RBD region (675 bp nucleotides, 224 amino acids; corresponding to the 319~541 amino acid region in the total spike protein) under the *hsp65* promoter from *M. bovis* BCG was cloned into a pMV306 vector. The pMV306-RBD vector was electroporated into Mpg, and we obtained rMpg-RBD strains.

**Figure 2:**
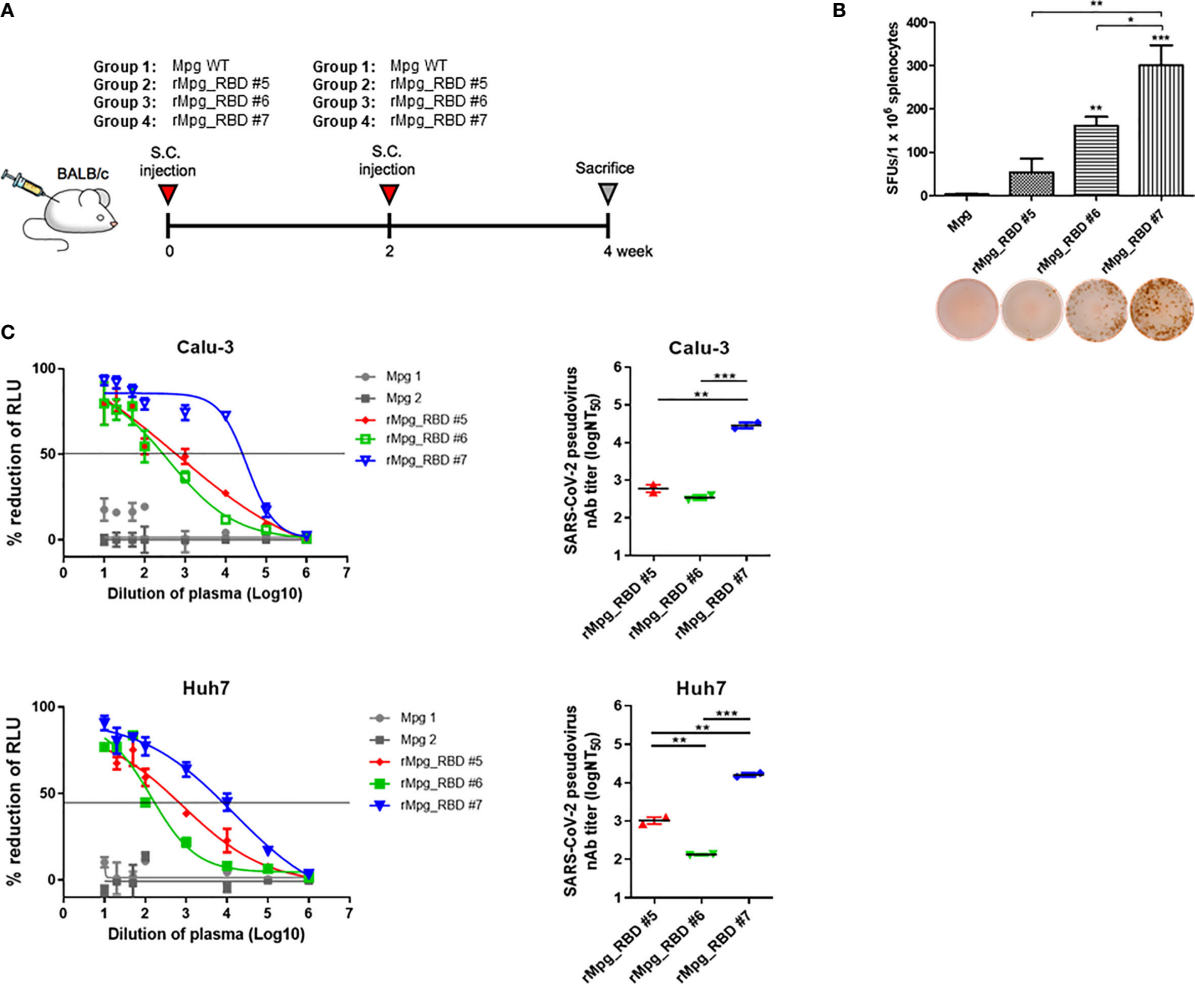
Selection of an rMpg-RBD-7 strain as a vaccine candidate eliciting strong RBD- or S1-specific immune responses in vaccinated mice. **(A)** Schematic mouse immunization schedule to select a vaccine candidate strain from three rMpg-RBD (-5, -6, and -7) strains. Wild-type Mpg (Mpg WT) or rMpg-RBD strains were subcutaneously injected twice at two-week intervals into BALB/c mice (*n* = 2~3). Two weeks after the final immunization, the mice were sacrificed, and their spleens and blood samples were collected for immunological analyses. **(B)** The IFN-γ secretion levels following an *in vitro* stimulation of splenocytes from mice vaccinated with two Mpg WT and three rMpg-RBD strains. Representative images of the ELISPOT membrane in each group are shown below the graph. **(C)** Pseudotyped SARS-CoV-2 neutralization assay in Calu-3 and Huh7 cells using sera from mice vaccinated with two Mpg wild-type and three rMpg-RBD strains. The RLU in Mpg was thought to be maximal virus infection in each dilution factor, and the neutralizing antibody titer (NT_50_) was calculated as RLU reduction rate compared to RLU in Mpg (control) using duplicate pseudotyped SARS-CoV-2 neutralization assays. The data are shown as the means ± SEM of mice (*n* = 2~3). Significant differences between each group are determined by one-way ANOVA and are shown as asterisks. *P < 0.05; **P < 0.01; and ***P < 0.001.

To examine the usefulness of rMpg in SARS-CoV-2 vaccines against viral infections, we generated rMpg-RBD strains expressing the SARS-CoV-2 receptor binding domain (RBD) using integrative *Mycobacterium-E. coli* shuttle vector pMV306 (30) (**Figure 1**). First, the rMpg-RBD-5, - 6 and -7 strains were randomly selected by colony PCR after the transformation of the *Mycobacterium-E. coli* shuttle vector pMV306-RBD into Mpg (**Supplementary Figure S1**). To select a final rMpg-RBD strain to elicit the strongest RBD or S1 subunit (S1) of the spike protein-specific immune response used as a vaccine candidate, a total of 5 strains, with two Mpgs as controls and three rMpg-RBD strains, rMpg-RBD-5, -6 and -7, being injected (1 × 10^6^ CFU/mouse) subcutaneously (S.C.) into mice (2 or 3 mice per group) twice at a 2-week interval as a two-dose vaccination model (**Figure 2A**). We found that all three rMpg-RBD strains could exert enhanced IFN-γ secretion against the SARS-CoV-2 S1 protein compared to the two control Mpg strains (**Figure 2B**). Furthermore, neutralization assays by a reporter lentivirus-based pseudovirus also indicated that all three rMpg-RBD strains elicited enhanced neutralizing activity against SARS-CoV-2 pseudovirus in Calu-3 and Huh-7 cells compared to Mpg wild type (**Figure 2C**). This finding suggests the potential use of rMpg-RBD strains as live SARS-CoV-2 vaccines. Since the rMpg-RBD-7 strain exerted the strongest cell-mediated immune responses and inhibited the cell entry capacity among the three rMpg-RBD strains, we ultimately selected the rMpg-RBD-7 strain for further evaluation in terms of vaccine efficacy in this study.

### rMpg-RBD-7 Strain Showed Stable RBD Expression in Bacteria and Infected DCs

**Figure 3:**
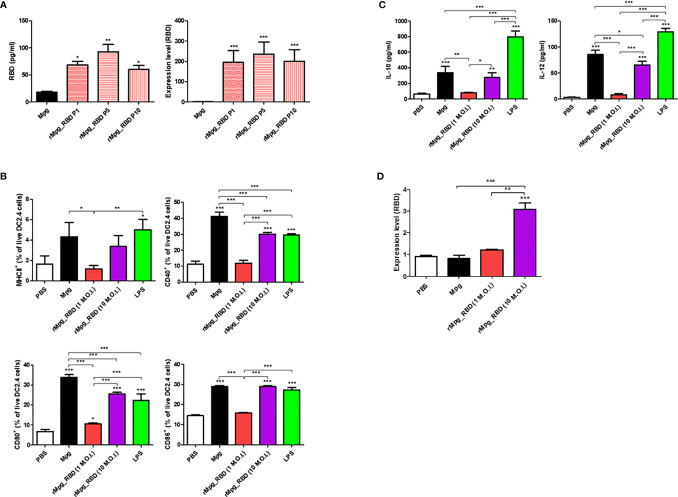
Stable expression of RBD in the rMpg-RBD-7 strain and in infected DC cells and its effect on DC maturation. **(A)** Confirmation of stable RBD expression in the rMpg-RBD-7 strain after passages of 1, 5, and 10 generations using RBD ELISA (left panel) and RT-qPCR (right panel). **(B)** Effect of the rMpg-RBD-7 strain on DC maturation in infected DC2.4 cells. DC2.4 cells were infected with Mpg WT (10 M.O.I.), the rMpg-RBD-7 strain (1 or 10 M.O.I.) or treated with LPS (1 μg/ml) for 24 h, stained with antibodies against DC maturation surface markers (MHCII, CD40, CD80, and CD86), and then analyzed by flow cytometry. **(C)** The cytokine expression levels of IL-10 and IL-12 in supernatants of DC2.4 cells infected with Mpg WT (10 M.O.I.), the rMpg-RBD-7 strain (1 or 10 M.O.I.) or treated with LPS (1 µg/ml) were detected by ELISA. **(D)** The mRNA expression level of RBD in rMpg-RBD-7-infected DC2.4 cells was confirmed by RT-qPCR. The data are shown as the means ± SEM of three independent experiments. Significant differences between each group are determined by one-way ANOVA and are shown as asterisks. ^*^ *P* < 0.05; ^**^ *P* < 0.01; ^***^ *P* < 0.001.

Next, we evaluated the stable RBD expression of the selected rMpg-RBD-7 as a vaccine candidate. To this end, we checked the RBD expression of the rMpg-RBD-7 strain after passages of 1, 5, and 10 generations *via* ELISA to detect the viral RBD proteins and perform real-time quantitative PCR (RT-qPCR) assays. Our data indicated that the rMpg-RBD-7 strain maintained RBD expression in bacteria for 10 generations at the translational or transcriptional levels, suggesting its stable RBD expression (**Figure 3A**). Next, we checked the effect of the rMpg-RBD-7 strain on the maturation and RBD expression of infected dendritic cells (DCs). To this end, the rMpg-RBD-7 strain infected DC2.4 cells and evaluated DC maturation markers *via* FACS analysis and RBD expression *via* RT-qPCR assay at 24 h.p.i. We found that although its expression was slightly lower than that of the Mpg strain, the rMpg-RBD-7 strain led to the increased expression of four DC maturation markers, MHC class II, CD40, CD80 and CD86, in infected DC2.4 cells in a dose-dependent manner (**Figure 3B**). A similar trend was also found in the IL-10 and IL-12 secretion in infected DC2.4 cells (**Figure 3C**). The rMpg-RBD-7 strain also led to increased RBD expression in a dose-dependent manner in infected DC2.4 cells (**Figure 3D**). However, some maturation markers such as CD40 and CD80 and IL-12 secretion in rMpg-RBD-7 strain infected DC2.4 cells were significantly lower than in wild type Mpg infected DC2.4 cells (**Figures 3B, C**), suggesting that rMpg-RBD-7 strain could compromise DC maturation capacity of wild type Mpg. Together, these data suggest that the rMpg-RBD-7 strain can maintain stable RBD expression in bacteria and infected DC cells, which can induce robust RBD-specific immune responses following RBD presentation to T cells in vaccinated mice.

### rMpg-RBD-7 Strain Led to Enhanced IgG or IgA Titers Against SARS-CoV-2 RBD in the Sera of Two-Dose Vaccinated Mice

**Figure 4:**
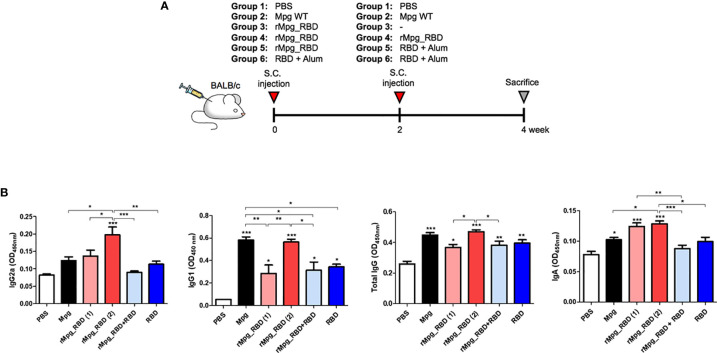
IgG (total IgG, IgG1 and IgG2a) and IgA titers against SARS-CoV-2 RBD induced by various injection conditions of the rMpg-RBD-7 strain in vaccinated mice. **(A)** Schematic schedule of mouse (*n* = 5) immunization with a two-dose injection (S.C.) of rMpg-RBD or RBD protein or a single rMpg-RBD injection. Mpg as a control was immunized with a two-dose injection (S.C.). Two weeks after the final immunization, the mice were sacrificed, and spleen, blood and BALF samples were collected for immunological analyses. **(B)** RBD-specific immunoglobulins (IgG2a, IgG1, total IgG and IgA) were detected by ELISA from sera or BALF samples from vaccinated mice. The data are shown as the means ± SEM of mice (*n* = 5). Significant differences between each group are determined by one-way ANOVA and are shown as asterisks. ^*^ *P* < 0.05; ^**^ *P* < 0.01; ^***^ *P* < 0.001.

Next, we evaluated the IgG or IgA titers against SARS-CoV-2 RBD induced by various injection conditions of the rMpg-RBD-7 strain in vaccinated mice. To this end, BALB/c mice were separated in 6 groups, and immunized S. C. 5 of this groups (PBS + PBS, Mpg + Mpg, rMpg-RBD + rMpg-RBD, rMpg-RBD + RBD protein with alum adjuvant, RBD protein with alum + RBD protein with alum) were immunized with as a two-dose vaccination mode with two-week intervals. The remain group was received a single injection of rMpg-RBD (**Figure 4A**). Two weeks after the last injection, serum and bronchoalveolar lavage fluid (BALF) samples were collected to detect the total IgG and IgG subtypes (IgG1 and IgG2a) and IgA, respectively. Our ELISA data showed that two injections of rMpg-RBD led to significantly elevated production of IgG2a and IgA against SARS-CoV-2-RBD in immunized mouse sera or BALF samples compared with that in other groups, suggesting that the two-dose vaccination model of rMpg-RBD could lead to enhanced Th1 skewed immune responses and a mucosal immune response against SARS-CoV-2-RBD (**Figure 4B**). Although generally similar results were also found in the total IgG or IgG1 titer against the SARS-CoV-2 RBD protein, as shown in the IgG2a and IgA titers, a two-dose vaccination of wild-type Mpg without RBD expression also led to higher titers of total IgG or IgG1, almost similar to a two-dose vaccination of rMpg-RBD (**Figure 4B**). This result is due to nonspecific production of IgG with a high binding affinity for RBD in the sera of vaccinated mice that were subjected to two-dose injections. Taken together, our data indicated that the two-dose vaccination model of rMpg-RBD led to enhanced IgG and IgA titers in the sera and BALF of vaccinated mice.

### rMpg-RBD-7 Strain Led to Enhanced Neutralizing Activity Against Pseudotyped or Live SARS-CoV-2 in the Sera of Two-Dose Vaccinated Mice

**Figure 5:**
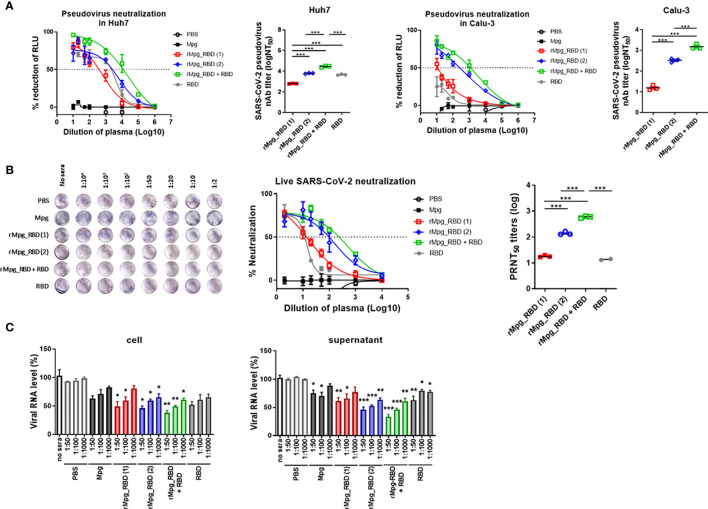
Two-dose vaccination of rMpg-RBD-7 or heterologous vaccination of rMpg-RBD-7 and RBD protein with alum exerts potent neutralizing activity against pseudotyped virus or live SARS-CoV-2. The potent neutralizing activity against SARS-CoV-2 in vaccinated mice was evaluated *via* neutralization assay against pseudotyped virus and live SARS-CoV-2. **(A)** Sera from the vaccinated mice were diluted and incubated with a reporter lentivirus-based pseudovirus for the neutralization assay. The RLU in Mpg was thought to be maximal virus infection in each dilution factor, and neutralizing antibody titer (NT_50_) was calculated as RLU reduction rate compared to RLU in Mpg (control) using duplicate pseudotyped SARS-CoV-2 neutralization assays in Calu-3 and Huh7 cells. **(B)** The sera from vaccinated mice were diluted and incubated with live SARS-CoV-2 for the plaque reduction neutralization test (PRNT) assay. The plaque formation in Mpg was thought to be maximal virus infection in each dilution factor, and fifty percent of the plaque reduction neutralizing antibody (PRNT_50_) titers against live SARS-CoV-2 were calculated as plaque reduction rate compared to Mpg (control) in Vero E6 cells. **(C)** The neutralization efficacy of the sera against the live SARS-CoV-2 RNA copy number in Vero E6 cells was determined by finding the mRNA expression level of RNA-dependent RNA polymerase in the cell and culture supernatants *via* RT-qPCR. The data are shown as the means ± SEM of mice (*n* = 2~3). Significant differences between each group are determined by one-way ANOVA and are shown as asterisks. ^*^ *P* < 0.05; ^**^ *P* < 0.01; ^***^ *P* < 0.001.

Additionally, the neutralizing activities of serum against SARS-CoV-2 virus infections induced by various injection conditions of the rMpg-RBD-7 strain in two-dose vaccinated mice of 6 groups were measured using a reporter lentivirus-based pseudotyped SARS-CoV-2 neutralization assay or a live virus-based plaque reduction neutralization test (PRNT) assay. First, we assessed the neutralizing activities in the mouse sera using a lentivirus-based reporter pseudovirus. The neutralization titers (NT_50_) were detected by tracking the reduction in the relative luciferase units (RLU) compared with the controls. Sera collected two weeks after the last immunization were used for the neutralizing assay. The pseudovirus was incubated with serial dilutions of mouse sera, and the sera-virus mixture was added to Huh7 and Calu-3 cells for 48 h. Consistent with our finding of the IgG2a and IgA titers, the pseudovirus neutralization assays showed that two-dose vaccination with rMpg-RBD-7 led to enhanced neutralizing activity against SARS-CoV-2 pseudovirus entry into Calu-3 and Huh-7 cells compared to two-dose vaccination with Mpg and single-dose vaccination with rMpg-RBD-7. However, in contrast to our result based on the antibody titer, a heterologous rMpg-RBD-7 vaccination as a prime injection and RBD protein with alum as a booster injection led to the highest neutralizing activity of the 6 groups, even compared to a two-dose rMpg-RBD-7 vaccination or a two-dose RBD protein vaccination with alum (**Figure 5A**). Second, for the live virus-based PRNT assay, ~120 pfu of live SARS-CoV-2 was incubated with serially diluted mouse sera, and the sera-virus mixture was used to infect Vero E6. Consistently, our PRNT assay indicated that higher PRNT_50_ titers were found in the two-dose injection of rMpg-RBD-7 or the heterologous injection of rMpg-RBD-7 and RBD protein with alum than in the other groups (**Figure 5B**), suggesting that they can effectively neutralize live SARS-CoV-2 infections. No antibody-dependent enhancement (ADE) effect was observed in the PRNT assay (**Supplementary Figure S2**). In addition, the mRNA levels of RNA-dependent RNA polymerase (RdRp) from live SARS-CoV-2 in the infected Vero E6 cell lysates or culture supernatants were the most strongly inhibited in the two-dose rMpg-RBD-7 vaccination or the heterologous rMpg-RBD-7 and RBD protein vaccine with alum compared to other groups using the sera at different dilutions (1:100, 1:1000 and 1:10000) in a dose-dependent manner (**Figure 5C**). These results revealed that the neutralizing activity of sera against live SARS-CoV-2 was the highest in the heterologous rMpg-RBD-7 and RBD protein vaccination with alum, with two-dose injection of rMpg-RBD-7 being the second highest.

### rMpg-RBD-7 Strain Led to an Enhanced Cell-Mediated Immune Response Specific to SARS-CoV-2 RBD in Two-Dose Vaccinated Mice

**Figure 6:**
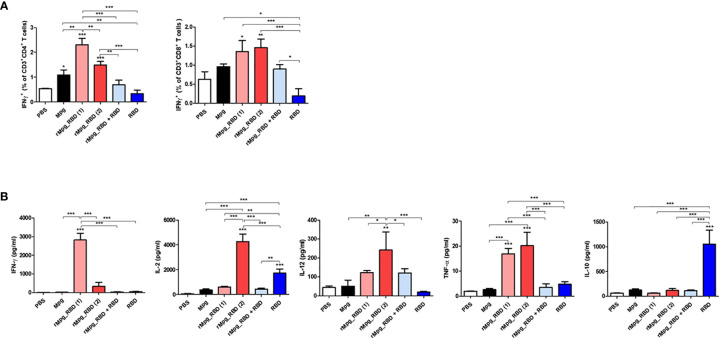
rMpg-RBD-7 exerts potent cell-mediated immune responses against SARS-CoV-2 RBD. **(A)** Comparison of IFN-γ secreting CD4 or CD8 T cell responses induced by rMpg-RBD or RBD protein immunized mice groups using flow cytometry. Splenocytes from immunized mice were stimulated *in vitro* with SARS-CoV-2 RBD protein (5 μg/ml) for 24 h and stained with antibodies against CD3, CD4, CD8 and IFN-γ to detect IFN-γ-secreting CD4 and CD8 T cells. **(B)** Splenocytes from immunized mice were stimulated with SARS-CoV-2 RBD protein (5 μg/ml) for 3 days. The cytokine expression levels of IFN-γ, IL-2, IL-12, TNF-α and IL-10 were detected by ELISA. The data are shown as the means ± SEM of mice (*n* = 5). Significant differences between each group are determined by one-way ANOVA and are shown as asterisks. ^*^ *P* < 0.05; ^**^ *P* < 0.01; and ^***^ *P* < 0.001.

Next, we also characterized the cell-mediated immune (CMI) response and induction of systemic cytokines in response to vaccination under various injection conditions of the rMpg-RBD-7 strain (**Figure 4A**). Splenocytes were harvested from mice at two weeks post-immunization in 6 groups, and flow cytometry was applied to splenocytes subjected to inoculation with SARS-CoV-2 RBD proteins (5 μg/ml) for 24 h. Our data demonstrated that the frequencies of IFN-γ-releasing CD4^+^ and CD8^+^ T cells were consistent with previous reports showing that the classical subunit protein vaccination protocol with alum adjuvants could lead to a poor CMI response due to a skewed Th2-type immune response (31, 32) (**Figure 6A**). In general, the profile of cytokines secreted from splenocytes indicated a similar result was obtained by the above flow cytometry-based activated T cell frequencies. The proinflammatory or Th1-type cytokines TNF-α, IFN-γ, IL-2, and IL-12 were profoundly induced by the RBD protein in the single- or two-dose rMpg-RBD-7 injection group (**Figure 6B** and **Supplementary Figure S3**). However, IL-10, an immunosuppressive cytokine, was profoundly increased in the two-dose injection group of RBD protein with alum compared to the other groups, suggesting a risk from RBD vaccinations with alum alone (**Figure 6B**). Taken together, our data demonstrated that irrespective of single- or two-dose injection, rMpg-RBD-7 vaccination led to a strong RBD-specific CMI response.

### Live rMpg-RBD-7 *Versus* HK-rMpg-RBD Led to Enhanced Cell-Mediated Immune Responses in Single Vaccination Mice

**Figure 7:**
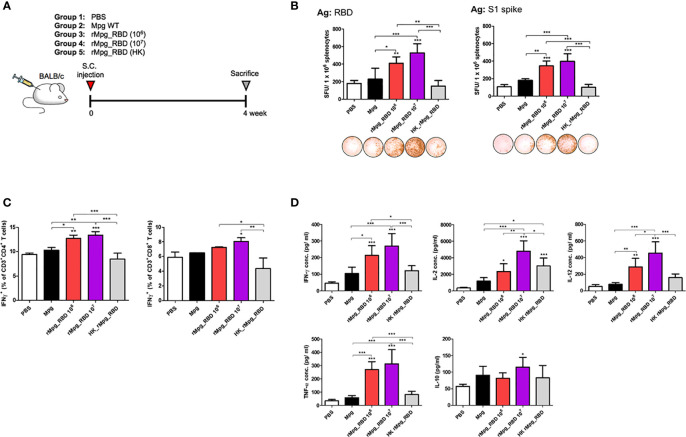
Live rMpg-RBD-7, but not HK-rMpg-RBD, exerts potent cell-mediated immune responses against SARS-CoV-2 RBD. **(A)** Schematic schedule of mouse (*n* = 5) immunization with live- or heat-killed rMpg-RBD single injection. Two weeks after the final immunization, the mice were sacrificed, and spleen, blood and BALF samples were collected for immunological analyses. **(B)** IFN-γ secretion was detected following the *in vitro* stimulation of splenocytes from immunized mice. Representative images of the ELISPOT membrane in each group are shown below the graph. **(C)** IFN-γ-secreting CD4 or CD8 T cells from immunized mice were compared by flow cytometry. **(D)** The cytokine expression levels of IFN-γ, IL-2, IL-12, TNF-α and IL-10 from *in vitro* stimulated cell culture supernatants were detected by ELISA. The data are shown as the means ± SEM of mice (*n* = 5). Significant differences between each group are determined by one-way ANOVA and are shown as asterisks. ^*^ *P* < 0.05; ^**^ *P* < 0.01; and ^***^ *P* < 0.001.

Next, we explored the vaccine potential against SARS-CoV-2 infection between live rMpg-RBD and heat-killed rMpg-RBD (HK-rMpg-RBD) in a single S.C. vaccination model of 5 groups, PBS, Mpg, live rMpg-RBD (1.0 × 10^6^ or 1.0 × 10^7^ CFU) and HK-rMpg-RBD (1.0 × 10^6^ CFU) (**Figure 7A**). In the IFN-γ ELISPOT assay, live rMpg-RBD led to an enhanced frequency of IFN-γ secreting cells in a CFU-dependent manner compared to Mpg or HK-rMpg-RBD, irrespective of the protein type, RBD or S1 protein (**Figure 7B**). Our flow cytometry data also showed that live rMpg-RBD *versus* Mpg or HK-rMpg-RBD led to an increased IFN-γ-releasing CD4 or CD8 T cell population in a CFU-dependent manner (**Figure 7C**). Cytokine profiles from the splenocytes of vaccinated mice also produced similar results to those found in ELISPOT and flow cytometry assays. The proinflammatory or Th1-type cytokines TNF-α, IFN-γ, and IL-12 were profoundly induced by RBD protein in the live Mpg-RBD group *versus* the Mpg or HK-rMpg-RBD group in a CFU-dependent manner by RBD stimulation. For IL-2 production, a similar level of 1.0 × 10^6^ CFU was found between live rMpg-RBD and HK-rMpg-RBD. In IL-10 production, a significant difference between the three strains, Mpg, live rMpg-RBD and HK-rMpg-RBD, of the same 1.0 × 10^6^ CFU was not found (**Figure 7D** and **Supplementary Figure S4**). Taken together, our data suggest that live rMpg-RBD, but not HK-rMpg-RBD, can lead to an enhanced CMI response against SARS-CoV-2 RBD in a single vaccination model.

### Live rMpg-RBD-7 Strain *Versus* HK-rMpg-RBD Led to Enhanced Neutralizing Antibody Production in Sera From Single Vaccination Mice

**Figure 8:**
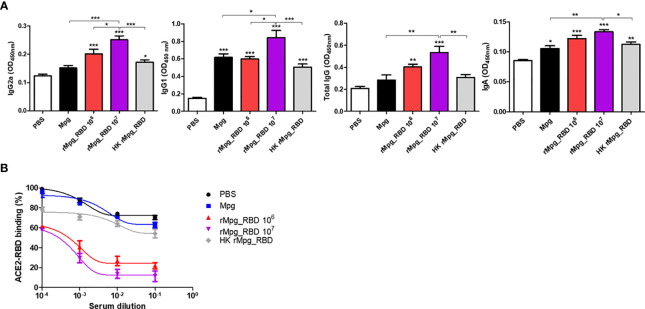
Live rMpg-RBD-7, but not HK-rMpg-RBD, exerts potent neutralizing antibody production against SARS-CoV-2 RBD. **(A)** RBD-specific immunoglobulins (IgG2a, IgG1, total IgG and IgA) were detected by ELISA from sera or BALF samples from immunized mice. **(B)** Inhibition of ACE2-RBD binding by serum samples from immunized mice was detected by an ACE2-RBD binding assay kit. The results were plotted with a nonlinear regression curve fit using the PRISM program. The data are shown as the means ± SEM of mice (*n* = 5). Significant differences between each group are determined by one-way ANOVA and are shown as asterisks. ^*^ *P* < 0.05; ^**^ *P* < 0.01; and ^***^ *P* < 0.001.

Next, to investigate the potential of the live rMpg-RBD-7 strain to engage in neutralizing antibody production in the vaccinated mice, we first checked the RBD-specific IgG production (IgG1, IgG2a, and total IgG) between 5 groups of vaccinated mice subjected to a single round of injection. Our ELISA data indicated that the live rMpg-RBD-7 strain led to increased IgG2a and total IgG titers in a CFU-dependent manner compared to Mpg or HK-Mpg-RBD. In the IgG1 titer, significant difference between three strains, Mpg, live Mpg-RBD and HK-rMpg-RBD, of the same 1.0 × 10^6^ CFU was not found (**Figure 8A**). To check the neutralizing activity of antibodies produced in vaccinated sera, ELISAs were conducted using the binding inhibition of RBD and ACE2 proteins by vaccinated sera. Our data indicated that the live rMpg-RBD-7 strain led to the increased inhibition of the interaction between RBD and ACE2 compared to Mpg or HK-Mpg-RBD. HK-Mpg-RBD led to a very marginal effect on binding inhibition compared to Mpg (**Figure 8B**). Together, our data suggest that live rMpg-RBD, but not HK-rMpg-RBD, can lead to enhanced neutralizing antibody production against SARS-CoV-2 RBD in a single vaccination model.

## Discussion

The recent emergence of new variants of SARS-CoV-2, including lineage B.1.1.7, first identified in the UK and lineage B.1.351, first identified in South Africa at the end of 2020, and their rapid worldwide expansion poses a significant threat to public health (33). Particularly, B.1.351 variants with two additional substitutions in RBD, a major target of vaccine, namely, K417N and E484K, can compromise the neutralizing activity of currently used vaccines by inhibiting the interaction of RBD with antibodies directed to ACE2-binding Class 1 and the adjacent Class 2 epitopes (34, 35). Therefore, due to these concerns about SARS-CoV-2 variants that are partially resistant to antibody defenses, the development of new vaccines harnessing T cell-mediated immune responses that are to be less affected by these mutated variants is in demand (36).

In this study, for the first time, we used live Mpg strains as a delivery vector of SARS-CoV-2 vaccine, and it is a slow-growing mycobacterium that is safe in an *in vivo* mice system and has the capacity to elicit robust T cell or humoral immune responses against loaded target antigens as indicated in our previous reports (24, 26). In addition to their adjuvant trait-enhancing vaccine efficacy, mycobacterial strains, including BCG, can also exert a heterologous vaccine effect, the so-called trained immunity based on the memory function of innate immune cells, of which vaccination can protect unrelated pathogens such as other respiratory viruses (37, 38). In fact, several studies have reported the merit of prior BCG vaccination in reducing COVID-19 patients or their disease severity (39–41), suggesting that the use of rMpg-RBD-7 could provide heterotypic vaccine immunity by trained immunity as well as RBD-specific homotypic vaccine immunity in vaccinated individuals. Our data showed that the Mpg strain without RBD expression could exert a significant increase in binding IgG to the RBD protein with a neutralizing capacity in the sera of the two-dose vaccination protocol (**Figure 5**), suggesting a partial induction of heterotypic vaccine immunity by Mpg.

Our data clearly demonstrated that even a single dose vaccination of rMpg-RBD-7 vaccination led to enhanced T cell-mediated immune responses against SARS-CoV-2 RBD (increased activated T cell frequencies or enhanced inflammatory or Th1 cytokine production from the splenocytes of vaccinate mice) compared to two doses of RBD protein with alum vaccination (**Figure 6**), suggesting the superiority of rMpg-RBD-7 vaccination over a classical RBD protein vaccination protocol in eliciting an RBD-specific CMI response. Notably, two-dose vaccinations with RBD protein and alum produced profound IL-10 secretion from the splenocytes of vaccinated mice, a signature of the immune suppressive response (42) (**Figure 6B**), suggesting that an RBD protein subunit vaccination could lead to a Th2 skewed immune response perhaps because of the alum adjuvant.

In general, the major drawback of using bacterial vector-based vaccines against viral infections is that most bacteria lack posttranslational modification systems, such as the glycosylation of viral proteins; for example, glycosylated SARS-CoV-2 RBD compared to eukaryotic expression systems (43). We sought to check the glycosylated status of the expressed RBD protein within rMpg-RBD-7 *via* Western blotting analysis. However, we failed to detect the expressed RBDs in rMpg-RBD-7 by Western blotting despite repeated experiments (data not shown), perhaps due to a lack of sensitivity or specificity of the anti-RBD antibodies used here. Nevertheless, we checked the stable expression of RBD protein or mRNA within the rMpg-RBD-7 strain by ELISA or q-PCR assay (**Figure 3A**). Irrespective of the nature of the expressed RBD, we found that two-dose vaccination with rMpg-RBD-7 can induce a significant increase in IgG binding to the RBD protein with a neutralizing capacity in the sera of the prime-booster vaccination protocol (**Figure 5**), even compared to a two-dose vaccination with glycosylated RBD protein with alum, suggesting that despite the nonglycosylated form of RBD in the former, the enhanced CD4 T cell-mediated immune response by the former vaccination could compensate for the reduced humoral immune response caused by the lack of glycosylation.

A heterologous vaccine protocol, combined with two different vaccine modules in prime and booster injections, reportedly elicits better protective effects in animal models of vaccines for various pathogens, including *M. tuberculosis* (44) or HIV-1 (45). Our data indicated that using a heterologous injection of rMpg-RBD-7 as a prime injection and RBD protein with alum as a booster can induce enhanced neutralizing activity in the sera of vaccinated mice compared to a two-dose injection of RBD protein with alum, or even to a two-dose injection of rMpg-RBD-7 protein (**Figure 5**), suggesting the feasibility of rMpg-RBD-7 in heterologous vaccine approaches. However, this vaccination can induce a lower level of T cell-mediated immune responses compared to single- or two-dose injection of rMpg-RBD-7, perhaps due to the Th2 skewing effect of the alum adjuvant used in prime vaccines (**Figure 6**). Notably, although the RBD binding IgG titers were significantly higher in two dose injections of rMpg-RBD-7 than in heterologous injections of rMpg-RBD-7 with RBD protein, even the virus neutralizing activity is higher in the latter than in the former vaccine (**Figure 4B**). This incongruent result may be due to a disparity in the ratio of neutralizing and non-neutralizing antigen-specific antibodies between the two vaccinated groups. Two-dose injections of rMpg-RBD-7 seem to produce increased non-neutralizing, antigen-specific antibodies as well as neutralizing antibodies. Given that non-neutralizing antigen-specific antibodies clearly have the potential to contribute to vaccine efficacy *via* complement activation (46), this result suggests an additional merit for two-dose injections of rMpg-RBD-7 as a SARS-CoV-2 vaccine.

Our data indicated that a single injection of rMpg-RBD-7 can induce a robust CMI response against the SARS-CoV-2 RBD, which is similar to or even higher than that induced by a two-dose injection of rMpg-RBD-7 (**Figure 6**), but RBD antigen-specific neutralizing antibodies were more enhanced after a two-dose injection (**Figure 5**). In addition, given the previous finding that the two-dose vaccine protocol is less pronounced in the reduction in antibody neutralization to various types of variants than single-dose vaccination (47), the two-dose vaccine protocol of rMpg-RBD-7 is likely to lead to a more protective vaccine effect than the single-dose protocol.

Lastly, we found that only the live version of rMpg-RBD-7, but not the HK strain, could induce CMI and a humoral immune response against SARS-CoV-2 RBD in a single-dose injection protocol (**Figures 7** and **8**). In particular, RBD antigen-specific neutralizing antibodies were rarely induced by HK Mpg-RBD-7 (**Figure 8B**), suggesting its poor protective role against SARS-CoV-2 infections.

The present study has an issue to be addressed. In this study, we did not evaluate the live SARS-CoV-2 neutralizing effect using heat-inactivated mouse serum. However, to eliminate non-specific inhibition effect by the complement, heat-inactivation (56°C, 30 min) of serum prior the assay was needed (48). 

**Figure 9:**
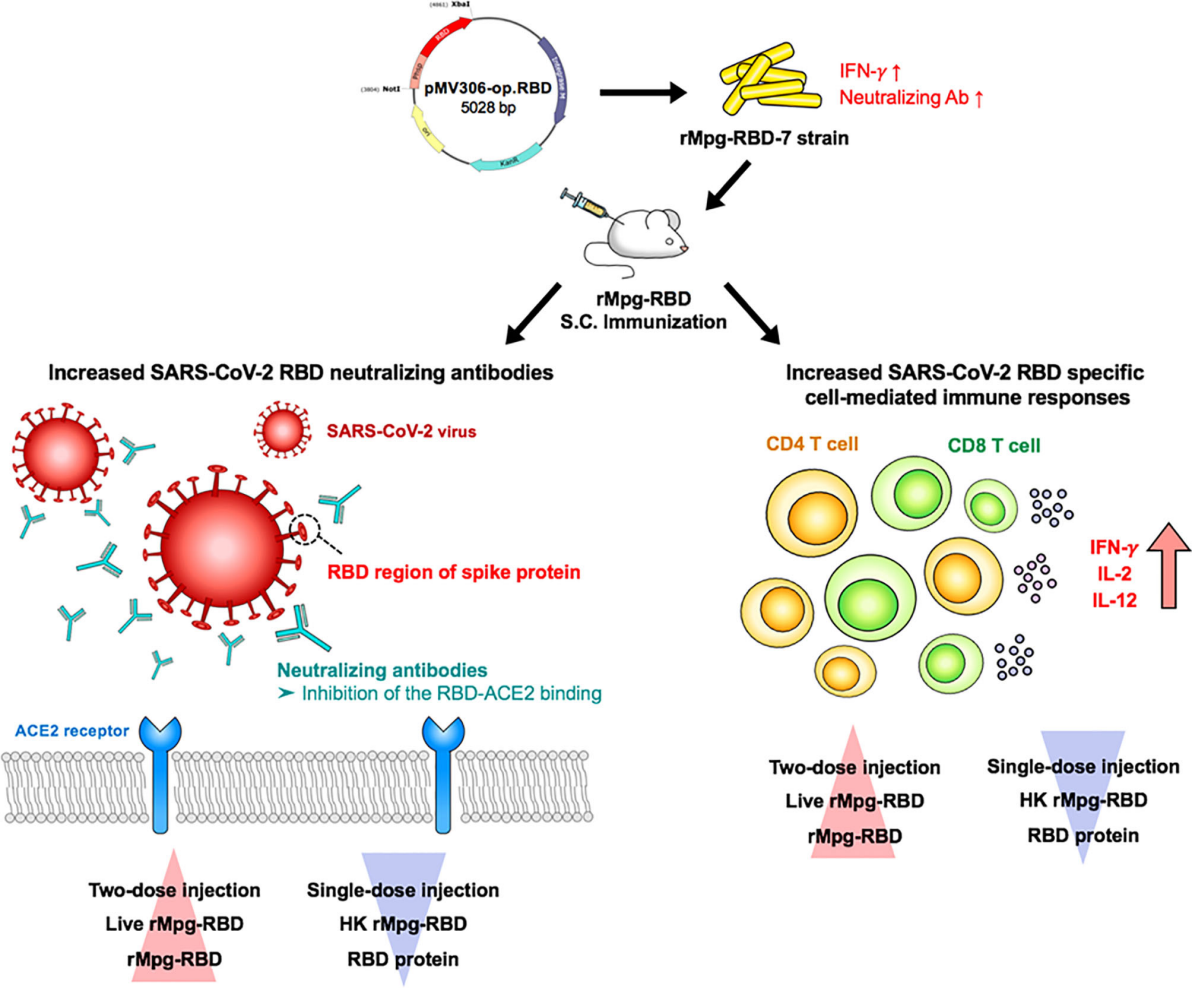
Schematic representation supporting the potential of the live rMpg-RBD-7 strain as a novel vaccine candidate against SARS-CoV-2 infection. We first selected an rMpg-RBD-7 strain showing strong CMI responses and neutralizing antibody production against SARS-CoV-2 infection *via* an *in vivo* mouse experiment. We found that vaccinating with the live rMpg-RBD-7 strain, particularly the two-dose vaccination, but not the HK strain, led to an enhanced anti-SARS-CoV-2 immune response in a mouse model. These results suggest the feasibility of the rMpg-RBD strain as a novel live vaccine against SARS-CoV-2 infection.

In conclusion, our data showed that live rMpg-RBD can lead to an enhanced CMI response and humoral immune responses against SARS-CoV-2 infections in mice subjected to single- or two-dose vaccination, highlighting its feasibility as a novel vaccine candidate for SARS-CoV-2 (**Figure 9**).

## Data Availability Statement

The original contributions presented in the study are included in the article/**Supplementary Material**. Further inquiries can be directed to the corresponding author.

## Ethics Statement

All the animal experiments were performed in accordance with the recommendations specified in the institutional guidelines, and the protocol was approved by the Institutional Animal Care and Use Committee (IACUC; approval No. SNU-200525-3) of the Institute of Laboratory Animal Resources at Seoul National University.

## Author Contributions

Bu-JK designed the research. By-JK, HJ, HS, and M-HL performed and analyzed overall experiments and wrote the manuscript. HS supported pseudovirus work. Bu-JK supervised overall experiments and wrote the manuscript. By-JK, HJ, and HS equally contributed to this study. All authors contributed to the article and approved the submitted version.

## Funding

This research was supported by Raphas Co Ltd (Grant No. 800-20200387) and by a grant of the Korea Health Technology R&D Project through the Korea Health Industry Development Institute (KHIDI), funded by the Ministry of Health & Welfare, Republic of Korea (Grant No. HV20C0147). The funder was not involved in the study design, collection, analysis, interpretation of data, the writing of this article or the decision to submit it for publication.

## Conflict of Interest

The authors declare that the research was conducted in the absence of any commercial or financial relationships that could be construed as a potential conflict of interest.

## Publisher’s Note

All claims expressed in this article are solely those of the authors and do not necessarily represent those of their affiliated organizations, or those of the publisher, the editors and the reviewers. Any product that may be evaluated in this article, or claim that may be made by its manufacturer, is not guaranteed or endorsed by the publisher.
