# Supplementary material for: Recombinant Mycobacterium paragordonae Expressing SARS-CoV-2 Receptor-Binding Domain as a Vaccine Candidate Against SARS-CoV-2 Infections
Source: Front Immunol. 2021 Aug 27;12:712274. doi: 10.3389/fimmu.2021.712274 (PMC8432291; doi:10.3389/fimmu.2021.712274)
Supplement: Supplementary file 1 [file DataSheet_1.docx]

Supplementary Material

# Supplementary Figures


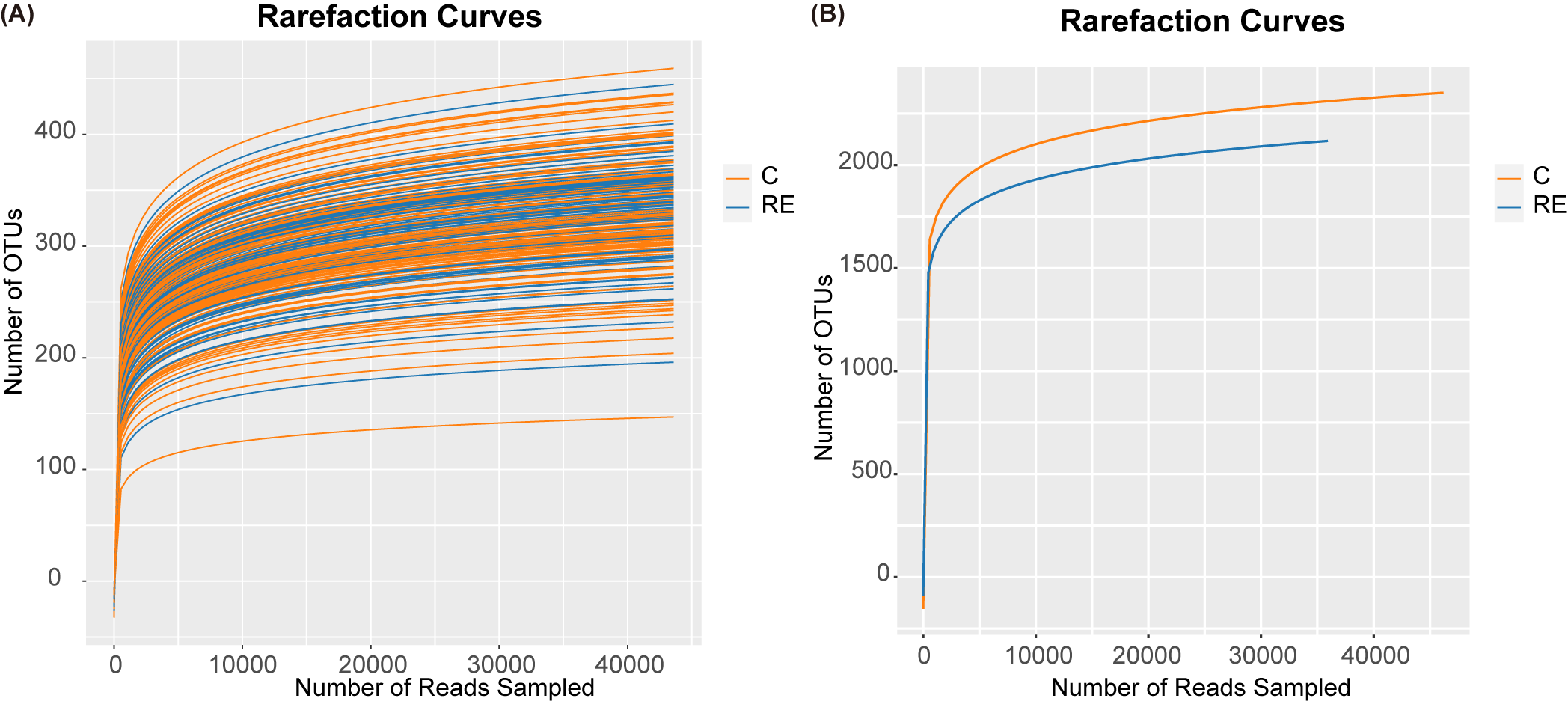


**Supplementary Figure 1.** Rarefaction curves of the 192 oral samples.


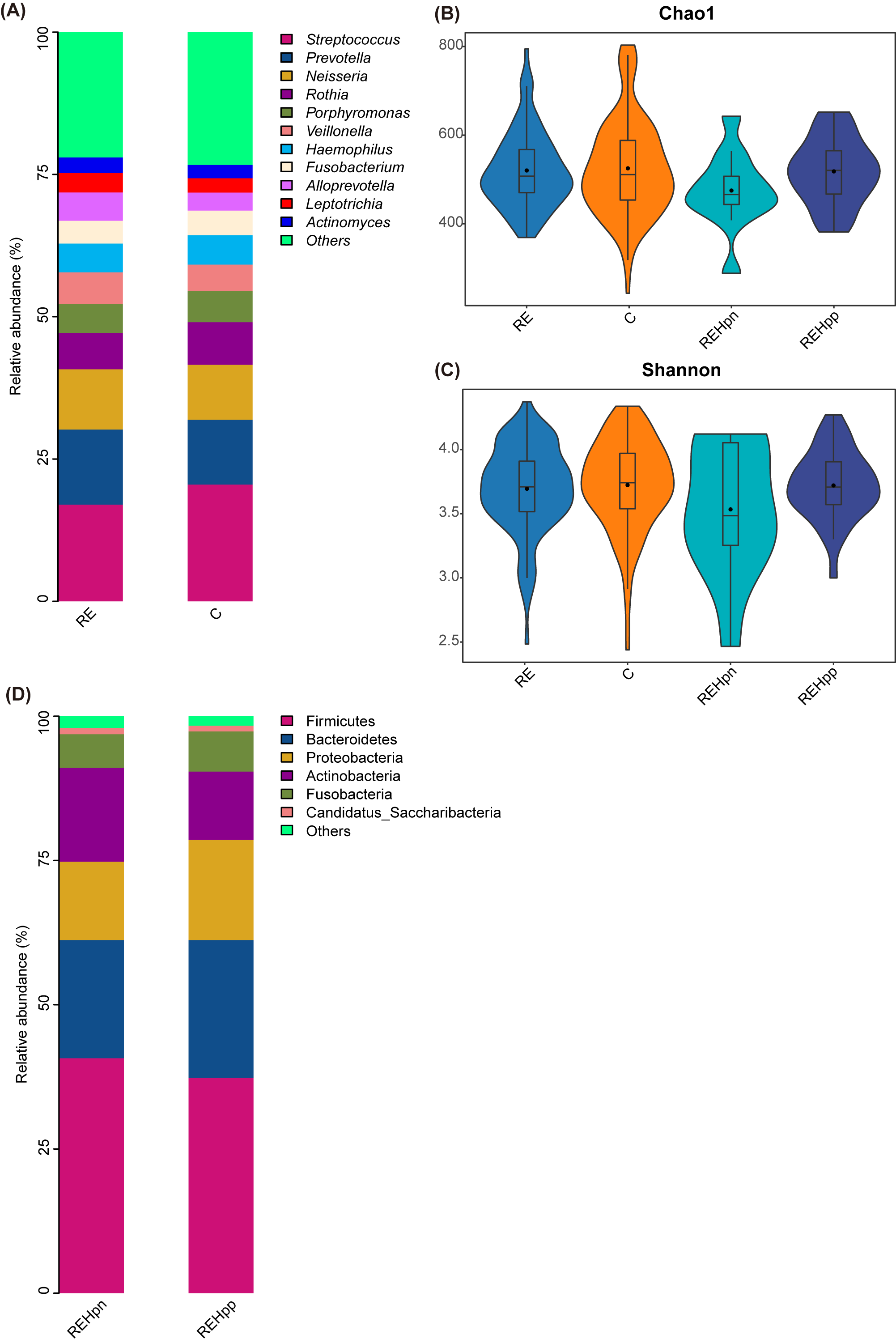


**Supplementary Figure 2.** Oral microbiota composition. (A) Comparison of the oral microbiota composition between the reflux esophagitis (RE) and the healthy groups at the genus level. (B-C) The influence of *Hp* infection on the oral microbiota of reflux esophagitis patients. The alpha diversity of the RE group was not different from that of the C, REHpn, and REHpp groups, *P* > 0.05. (D) Comparison of oral microbiota composition between the REHpn and REHpp groups at the phylum level.
